# Supplementary material for: Fever in Children with Cancer: Pathophysiological Insights Using Blood Transcriptomics
Source: Int J Mol Sci. 2025 Jul 24;26(15):7126. doi: 10.3390/ijms26157126 (PMC12346811; doi:10.3390/ijms26157126)
Supplement: Supplementary file 1 [file ijms-26-07126-s001.zip › ijms-3693508-supplementary.pdf]

Supplementary Material

Table S1. Bloodstream bacteria identified on day 1 in febrile children with cancer.

Day 1

Febrile samples	Pathogen 1	Pathogen 2	Pathogen 3
1	*Pseudomonas aeruginosa*	*Elizabethkingia meningoseptica*	
2	*Pseudomonas aeruginosa*		
3	*Staphylococcus aureus*	Coagulase-negative staphylococci	
4	*Enterobacter cloacae*		
5	*Streptococcus viridans*		
6	*Klebsiella pneumoniae*	Coagulase-negative staphylococci	
7	*Acinetobacter pittii*		
8	*Staphylococcus aureus*		
9	*Enterococcus faecium*		
10	*Escherichia coli*		
11	*Escherichia coli*		
12	*Staphylococcus aureus*		
13	*Stenotrophomonas maltophilia*	Coagulase-negative staphylococci	
14	*Staphylococcus aureus*		
15	*Streptococcus viridans*		
16	*Pseudomonas aeruginosa*		
17	*Pseudomonas aeruginosa*	Coagulase-negative staphylococci	
18	*Streptococcus viridans*		
19	*Streptococcus viridans*		
20	*Enterobacter cloacae*		
21	*Streptococcus viridans*	*Pseudomonas koreensis*, *Neisseria subflava*	Coagulase-negative staphylococci
22	*Klebsiella pneumoniae*	*Stenotrophomonas maltophilia*, *Citrobacter braakii*	
23	*Escherichia coli*		
24	*Escherichia coli*	*Klebsiella pneumoniae*	
25	*Escherichia coli*	*Enterococcus faecium*	*Rothia mucilaginosa*
26	*Staphylococcus aureus*		
27	*Enterococcus faecalis*	*Enterococcus gallinarum*	

Table S2. Bloodstream bacteria identified on day 2 in febrile children with cancer.

Day 2

Febrile episode	Pathogen 1	Pathogen 2	Pathogen 3
1	*Pseudomonas aeruginosa*		
2	*Pseudomonas aeruginosa*		
3	*Streptococcus viridans*	*Mixta calida*	
4	*Pseudomonas aeruginosa*		
5	*Staphylococcus aureus*		
6	*Enterobacter cloacae*		
7	*Streptococcus viridans*		
8	*Escherichia coli*		
9	*Pseudomonas aeruginosa*	*Escherichia coli*	*Klebsiella pneumoniae*
10	*Escherichia coli*		

Table S3. The top 20 up- and downregulated genes on day 2 in febrile children with cancer and bacteremia compared to those with unexplained fever and low CRP.

Gene symbol	Gene name	Log2FC	padj	Function
NSUN3	NOP2/Sun RNA Methyltransferase	3.32	<0.0001	RNA binding ad methyltransferase activity.
PCMTD1	Protein-L-Isoas-partate	2.82	<0.0001	Protein methylation.
SLF1	SMC5-SMC6 Complex Localization Factor 1	3.41	<0.0001	DNA damage response.
MAN1A2	Mannosidase Alpha Class 1A Member 2	2.99	<0.0001	Glycosylase.
MACO1	Macoilin 1	3.45	<0.0001	Actin and microtubule binding activity. Regulation of neuronal activity.
SLC39A8	Solute Carrier Family 39 Member 8	3.17	<0.0001	Zinc transporter.
SEC62	SEC62 Homolog, Preprotein Translocation Factor	3.05	<0.0001	Mediates post-translational transport of precursor polypeptides across endoplasmic reticulum.
AKAP7	A-kinase Anchoring Protein 7	3.93	0.0001	Targets cAMP-dependent protein kinase to the cellular membrane or cytoskeletal structures.
TOP1	DNA Topoisomerase I	2.12	0.0002	Controls and alters the topologic states of DNA during transcription.
MBNL2	Muscleblind Like Splicing Regulator 2	3.40	0.0002	Mediates pre-mRNA alternative splicing regulation.
UQCRB	Ubiquinol-Cytochrome C Reductase Binding Protein	2.82	0.0002	Hypoxia-induced angiogenesis through mitochondrial reactive oxygen species-mediated signaling.
FCHO2	FCH And Mu Domain Containing Endocytic Adaptor 2	3.99	0.0002	Protein binding activity. Clathrin coat assembly and endocytosis.
ZC3H6	Zinc Finger CCCH-Type Containing 6	3.63	0.0002	Enables RNA binding activity and metal ion binding activity.
CTSG	Cathepsin G	-6.80	0.0082	Trypsin- and chymotrypsin-like specificity. Antibacterial activity against Gram-negative and -positive bacteria.
SNN	Stannin	-1.61	0.0223	Enables metal ion binding activity. Predicted to be involved in response to toxic substance.
KATNB1	Katanin Regulatory Subunit B1	-1.61	0.0260	Participates in a complex which severs microtubules in an ATP-dependent manner.
MAN2C1	Mannosidase Alpha Class 2C Member 1	-2.08	0.0300	Glycosylase. Oligosaccharide catabolic process.

MPO	Myeloperoxidase	-4.63	0.0444	Part of the host defense system of polymorphonuclear leukocytes. Responsible for microbicidal activity against a wide range of organisms.
ELANE	Elastase, Neutrophil Expressed	-5.38	0.0444	Modifies the functions of natural killer cells, monocytes and granulocytes.
ZER1	Zyg-11 Related Cell Cycle Regulator	-1.99	0.0480	Subunit of an E3 ubiquitin ligase complex that may be involved in meiosis.

Table S4. The top 20 up- and downregulated genes on day 1 in febrile children with cancer and unexplained fever and high CRP compared to those with bacteremia.

Gene symbol	Gene name	Log2FC	padj	Function
ATP8	Mitochondrially Encoded ATP Synthase Membrane Subunit 8	2.88	<0.0001	Contributes to proton-transporting ATP synthase activity, rotational mechanism.
SERF2	Small EDRK-Rich Factor 2	2.62	<0.0001	Involved in protein destabilization. Positive regulator of amyloid protein aggregation and proteotoxicity.
H4C3	H4 Clustered Histone 3	2.79	<0.0001	Core component of nucleosome. play a central role in transcription regulation, DNA repair, DNA replication and chromosomal stability.
ATP5F1E	ATP Synthase F1 Subunit Epsilon	2.21	<0.0001	Encodes a subunit of mitochondrial ATP synthase. Mitochondrial ATP synthase catalyzes ATP synthesis.
CLC	Charcot-Leyden Crystal Galectin	4.24	<0.0001	Regulates immune responses through the recognition of cell-surface glycans. Essential for the anergy and suppressive function of CD25-positive regulatory T-cells (Treg).
IFI27	Interferon Alpha Inducible Protein 27	4.52	<0.0001	Involved in type-I interferon-induced apoptosis.
SNRPD2	Small Nuclear Ribonucleoprotein D2 Polypeptide	2.11	<0.0001	Plays a role in pre-mRNA splicing.
ROMO1	Reactive oxygen Species Modulator 1	1.89	<0.0001	Induces production of reactive oxygen species (ROS) which are necessary for cell proliferation.
LINC00570	Long Intergenic Non-Protein Coding RNA 570	3.95	<0.0001	Long Intergenic Non-Protein Coding RNA gene.
UBL5	Ubiquitin Like 5	1.67	<0.0001	Plays a role in RNA processing and cellular stress responses.
CXCL1	C-X-C Motif Chemokine Ligand 1	-3.67	<0.0001	Chemotactic activity for neutrophils.
NT5DC3	5'-Nucleotidase Domain Containing 3	-2.41	0.0011	Predicted to enable 5'-nucleotidase activity. Located in mitochondrion. Part of receptor complex.
MYOZ3	Myozenin 3	-3.76	0.0012	Expressed in the skeletal muscle and belongs to the myozenin family. Members of this family function as calcineurin-interacting proteins that help tether calcineurin to the sarcomere of cardiac and skeletal muscle.
MGLL	Monoglyceride Lipase	-1.78	0.0013	Monoacylglycerol lipase (MAGL, MGL) is a hydrolase that is involved in endocannabinoid and triglyceride hydrolysis, lipid signaling.
TOGARAM2	TOG Array Regulator Of Axonemal Microtubules 2	-2.31	0.0016	Involved in microtubule cytoskeleton organization.
VSIG4	V-Set And Immunoglobulin Domain Containing 4	-2.79	0.0024	Phagocytic receptor, strong negative regulator of T-cell proliferation and IL2 production. Potent inhibitor of the alternative complement pathway convertases.

RAI14	Retinoic Acid Induced 14	-3.72	0.0030	Predicted to enable actin binding activity. Predicted to be involved in cell differentiation and spermatogenesis.
ZC3H12B	Zinc Finger CCCH-Type Containing 12B	-2.11	0.0034	Proinflammatory activation of macrophages. The exact function of this family member is unknown, but it is thought to function as a ribonuclease.
HSPG2	Heparan Sulfate Proteoglycan 2	-3.37	0.0037	Encodes perlecan protein which is a key component of the vascular extracellular matrix. Helps maintaining the endothelial barrier function. It is a potent inhibitor of smooth muscle cell proliferation and is thus thought to help maintain vascular homeostasis.
INPP5A	Inositol Polyphosphate-5-Phosphatase A	-1.57	0.0042	Mobilizes intracellular calcium and acts as a second messenger mediating cell responses to various stimulation.
